# People and Families Affected by Glycogen Storage Disease Type Ia: An Analysis of Narrative Accounts Written by Individuals Living with GSDIa and Their Caregivers

**DOI:** 10.36469/001c.131811

**Published:** 2025-04-03

**Authors:** Eliza Kruger, Hayley M. de Freitas, Iris Ferrecchia, Millie Gaydon, Andrew Lloyd

**Affiliations:** 1 Ultragenyx Pharmaceutical Inc., Novato, CA, USA; 2 Acaster Lloyd Consulting Ltd, London, UK; 3 University of Connecticut, Farmington, CT, USA

**Keywords:** glycogen storage disease type Ia, patient-reported outcome, narratives, quality of life, patient, caregiver

## Abstract

**Background:** Glycogen storage disease type Ia (GSDIa) is a rare inherited disorder that can lead to renal and hepatic complications, brain damage, and death. There is a lack of qualitative research describing the impact of GSDIa on individuals and their caregivers. **Objective:** This study was designed to describe the individual experiences of those affected by GSDIa through qualitative methods. **Methods:** Individuals with GSDIa and caregivers were recruited through two patient associations (US, UK). Participants were asked to write narratives about their experiences with GSDIa in their own words, with no set questions or topics. The resulting narrative accounts were analyzed thematically using a primarily inductive approach. Themes describing the impact of GSDIa on individuals and caregivers were identified. **Results:** Eight caregivers, three of whom had lost a child to GSDIa, and 11 individuals living with GSDIa provided written summaries of their experiences of life with the condition. Participants described symptoms, the burden of managing frequent cornstarch feeds, and the broader impacts of GSDIa. These included effects on daily activities, family, emotional and social functioning, romantic relationships, and educational/professional impacts. Participants also described challenges related to obtaining diagnosis and support, and the lack of GSDIa knowledge from healthcare professionals. **Discussion:** This study provided novel insights into the impact of GSDIa on individuals living with the condition and their caregivers. Participants reported a wide range of issues in their narratives and openly shared sensitive information about their experiences, which may not have been captured if they had taken part in a semi-structured interview format. **Conclusion:** The narrative method yielded a rich data set that provided details of the varied and individual experiences of GSDIa not captured by traditional quantitative methods or structured interviews. The narratives highlighted the unmet needs of both caregivers of and patients with GSDIa around cornstarch management, obstacles to proper treatment from healthcare professionals, and the absence of effective treatments. The open-ended narrative approach had advantages over standard qualitative methods to capture greater insight into individual experiences directly from participants in their own words.

## BACKGROUND

Glycogen storage diseases (GSDs) are a set of genetic disorders caused by a defective enzyme responsible for regulating the synthesis or degradation of glycogen.^1^ These rare and potentially very severe conditions may not be well understood in terms of the ways patients’ lives are affected. GSD type Ia (GSDIa) is categorized by a deficiency in glucose-6-phosphatase and is responsible for 80% of GSDI cases.^1^ GSDIa has a global incidence rate of 1 in 100 000.^2^

The most serious consequence of GSDIa is potentially life-threatening hypoglycemia. Poor metabolic control can increase the risk of developing brain damage, liver adenomas, chronic kidney disease, liver cancer, and osteoporosis.^3–6^ Many individuals experience hepatomegaly and renomegaly. People can experience frequent lethargy, overwhelming hunger, anemia,^3^ stunted growth, delayed puberty,^7^ and polycystic ovaries.^8^ While there is currently no cure for GSDIa, dietary measures can be used to manage the disease. These include consuming cornstarch and other dense carbohydrates every 1 to 4 hours to maintain blood glucose levels.^9,10^ These strictly timed feeds are demanding for both individuals living with GSDIa and their caregivers and can often lead to sleep disturbances.^11^ Parents of children with GSDIa report significantly elevated psychological stress and stress associated with caring for a child with a medical illness.^12^ However, the impact and burden of GSDIa and many other rare diseases is often misunderstood and poorly documented.

Different research approaches are used in health care to explore the patient experience of disease and treatment, and understand patient health-related beliefs, motivations, and behavior. Patient-reported outcome (PRO) measures are commonly used and are important for assessing the patient’s experience of a disease.^13,14^ However, PRO questionnaires commonly collect quantitative data through the administration of closed-ended questions that do not capture the context or reasons for patient responses. While some PROs include open-ended response questions (eg, allowing patients to describe their experiences in their own words), the fields are often limited and present challenges for the management and analysis of these data in PRO administration.^14^

Qualitative research methods, such as patient interviews and focus groups, encourage patients to describe their experience in their own words and capture data that is contextualized (ie, patients are prompted to describe and explain the factors that influence their perspectives and experiences).^15^ Semi-structured discussion guides are commonly used to conduct interviews and focus groups, often with practical limits on the number of topics and time allotted for each question, and topics of most interest to individual patients may not be prioritized in the discussion guide.^16^

Other types of qualitative research used in health care, such as narrative research, ask the patient to describe their lived experience of their disease in their own words without structure imposed on time or topic areas. Narrative research analyzes stories, diaries, journals, conversations, or other forms of unprompted dialogue or interaction between people.^15,17–19^ The method has been used in many areas of social science but is less commonly applied within health care.^19^ Narrative descriptions written by an individual or family member, detailing how a condition has affected their lives, provide a different method to describe personal experiences and impacts of a disease and treatments. The narrative approach allows participants to express themselves freely and describe their unique experiences in their own words rather than the discussion being directed or constrained by the researcher.^15^ This approach may allow participants to feel enabled to write about more personal aspects of their experience because they are not speaking directly to a researcher in an interview situation. Additionally, participants can write these narratives in their own time, making them less burdensome, enabling participants to consider in depth what they want to write about vs immediately answering an interview question. This method has been previously employed to capture similar data in other disease states.^20,21^

The present study was designed to explore the impact of GSDIa on people with the disease and their caregivers. An open-ended, narrative approach to data collection was used to capture the range of problems people experience in their own words, completed in their own time. Participants wrote narrative descriptions of how GSDIa affects their lives. These narrative accounts underwent qualitative analysis to provide an overall summary of the experiences of the impact and burden of GSDIa through the meaning and language that patients and caregivers used to describe it.

## METHODS

### Sample

Nineteen study participants were recruited through the Association for Glycogen Storage Disease (UK) and The Children’s Fund for Glycogen Storage Disease Research (US). Participants included adults diagnosed with GSDIa or adults who currently or have previously cared for someone with GSDIa. Participants were current residents of North America and the UK, had not participated in a clinical trial by the study sponsor, and had a proficient level of written English.

### Procedures

Participants were screened via telephone and, if they indicated interest in participating, proceeded to the informed consent stage. Written informed consent was obtained prior to interviews. Consenting participants then completed a sociodemographic background questionnaire and were sent instructions on the narrative task, which included examples of topics they might like to describe. Participants were free to write about any topic of their choosing and were not limited on how long the narrative could be. Participants had 3 weeks to return the narrative but were given more time if requested. A member of the Acaster Lloyd Consulting research team called each participant to discuss the narrative writing task and ensure participants understood the task. Participants’ narrative accounts were securely transferred and anonymized. Participants received a study remuneration of US$100/£75. Ethical approval was obtained by the Western Connexus Group Independent Review Board.

### Analysis

Participants’ written narratives were analyzed using thematic analysis, a qualitative method for identifying, analyzing, and reporting themes in qualitative data.^22^ The analysis aimed to identify concepts relating to the burden and impact of GSDIa, from the perspective of patients and caregivers. Given the open nature of the narrative task with no set topics, a primarily inductive approach was taken.

Data analysis was supported using MAXQDA,^23^ a software tool that assists with organizing qualitative data; the program does not automate any of the analysis process. A quarter of the transcripts were coded by two members of the research team to ensure consistency in the analysis. During the coding process, the constant comparative method, an iterative coding approach moving between narratives and new codes that emerge, was followed. The codes were then reviewed in detail to identify relevant concepts that were then grouped into themes to describe participants’ experience. Word clouds of the emotional impacts reported by participants were developed using the Janson Davies word cloud generator.^24^ The emotions described by participants were listed in the word cloud generator according to the number of times participants reported them overall. Words included in the word cloud were those used by participants. A thematic network analysis of participant symptoms and impacts was completed using Visual Tools in MAXQDA. The networks depict the proximity of codes and themes reported. When generating these figures, “close proximity” was defined as being within the same paragraph in the participant written narratives. Only the two most frequently reported symptoms and health-related quality-of-life impacts were input to the network analysis to focus on those considered most meaningful to participants. The networks were customized so that the size of the code circle would represent how frequently the codes were reported in the narratives and lines between codes to represent where codes were co-reported a minimum of 4 times. The networks were also tailored so that code circles located close in proximity show frequently co-reported codes as well as code circles of the same color depicting themes reported close in proximity in the participants’ narratives. Sociodemographic data were summarized using descriptive statistics.

## RESULTS

### Sample Characteristics

Narratives were captured from 11 individuals with GSDIa and 8 caregivers (**Table 1**). Cornstarch consumption ranged from 24 g/day to 360 g/day. Five participants living with GSDIa reported using glucose monitors. Additionally, participants reported using various medications to help manage their GSDIa.

**Table 1.** Study Sample Characteristics (N = 19)

**Characteristic**	**Participants With GSDIa (n = 11)**	**Caregivers (n = 8)**
Age (years)		
18-24	5	0
25-34	2	2
35-44	2	4
45-54	2	0
≥65	0	2
Sex		
Male/female ratio	5:6	1:7
Country of residence		
United Kingdom	3	3
North America	8	5
Employment status		
Employed	7	4
Student	3	0
Self-employed	1	1
Full-time homemaker/caregiver	0	3

Dependents of caregivers ranged in age from 3 to 25 years and older, including five children in school. One participant cared for two individuals with GSDIa; the rest of the caregiver sample cared for one individual with GSDIa. Six caregivers had other children without GSDIa. All but one caregiver stated they shared caregiving responsibilities but estimated that they were responsible for 50% to 80% of care. Three caregivers had lost a child to GSDIa.

### Main Findings from Narratives

The narrative data from participants provided a detailed description of life with GSDIa for individuals and caregivers. The narratives were 1 to 4 pages in length, except one that was 75 pages long. Although they wrote about similar experiences, participants presented their thoughts differently. Some participants presented their thoughts as an anecdotal story going into detail about a day or time of their life (eg, their diagnosis journey or moving away from home). Other participants presented their ideas as a topical list. The narrative data outlined below are described in the words of the participants, including some possible errors in terminology.

### Participant-Reported Symptoms of GSDIa

Participants described many different symptoms, including tiredness or lack of energy, short stature, and an enlarged liver. Participants also discussed experiencing dramatic blood sugar swings (**Table 2, Quote 1**).

**Table 2.** Participant Quotations

Quote 1	“Many of us with GSD have wild blood sugar swings. Like, we can be at 140 and in 40-60 minutes can fall 100 points, even with eating or having cornstarch like we normally would.” – P.12
Quote 2	“The fatigue, the medical routine and strict diet illustrate this unique relationship GSD patients and families have with time. We cannot forget the time, never. We are living clocks.” – P.13
Quote 3	“I have to wake up every 4 hours in order to consume cornstarch, which disrupts my sleep and stops my body from ever accessing important [rapid eye movement] sleep, so I am constantly exhausted and deprived of sleep.” – P.17
Quote 4	“It is imperative that you approve new treatments of GSD1A for safety […] Even if the treatment is not a cure, but purely extends my fasting capabilities by a matter of hours, the effect it will have on my entire well-being and life is all-encompassing.” – P.17
Quote 5	“My parents were told that I wouldn’t live past a year or two and to take me home and love me the best way they knew how for as much time as we had left. I defied those odds as I’m 42 now and living a fabulous, healthy life.” – P.01
Quote 6	“’You have what?’ followed by ‘I haven’t seen this since medical school.’” – P.12
Quote 7	“We have no idea how to treat you. You tell us what to do, and we’ll do it.” – P.12
Quote 8	“They did not give us a lot of hope […] they told us they get sick very often, they are admitted a lot to the hospital, don’t have normal growth, intellectual delays, may not walk until 5 years old, and to not talk a lot about [the] future.” – C.03
Quote 9	“On top of this, I had to go through it alone. Because there are only 5999 more people like me in the world, there is hardly a support system and no one who understands my struggles.” – P.11
Quote 10	“’I’m very happy my kids are healthy thanks to doctors, dieticians and a lot of people that have helped us during this journey” – C.03
Quote 11	“The smallest bug makes him sick… being sick almost always results in days in hospital.” – C.16
Quote 12	“I am never more than 10 hours from death […] It gives me extreme anxiety.” – P.17
Quote 13	“GSDIa is likely the root cause of all of my body image issues. I often feel self-conscious of my weight and my additional fat stores.” – P.17
Quote 14	“I don’t think people have any idea of how detrimental this is to a little kid. On birthdays, you have to say no to your own birthday cake” – C.15
Quote 15	“I got picked on a lot at school because of my size. I have heard every name in the book” – P.10
Quote 16	“All of my struggles were internal. Invisible.” – P.11
Quote 17	“I was hiding the truth because I was afraid no one would see me for myself anymore, that instead, everyone would treat me differently.” – P.05
Quote 18	“Also, there’s this other aspect called dating […] I’ve had folks say, ‘I can’t handle that’ and it’s done. It’s like wait, did I just become a monster? What just happened?” – P.12
Quote 19	“My mind never shuts off. My home was between worry and fear 24/7.”– C.18
Quote 20	“GSDIa vastly limits your career choices. Physical jobs are almost out of the question, due to my body’s constant demand for food and stable blood sugars.” – P.17
Quote 21	“He passed away […] due to complications of our disease. When I heard of his passing, I was so sad but even more afraid that one day, that’d be me.” – P.08

Abbreviations: GSD, glycogen storage disease; GSDIa, glycogen storage disease type Ia.

Other reported symptoms included aches and pains, heavy breathing, digestion issues, “stunted puberty,” sweating, fast heart rate, hunger, high triglycerides, high uric acid, and kidney stones. Caregivers described other symptoms not mentioned by patients themselves, including breathing difficulties, pallidity, seizures, gout, fever, constant hunger, and nausea and vomiting.

Several participants suggested their symptoms and their own response to treatment had changed over their lifetime. Participants also stated how they had developed new health problems such as fatty liver disease and diabetes.

### GSDIa Management

**Monitoring blood sugar levels and planning ahead**: Participants described having to be constantly vigilant of their blood sugar levels and often having to excuse themselves from situations to monitor these levels. Participants also discussed having to plan “meticulously” around their glucose regimens, which reduced spontaneity in their life.

**Eating and drinking schedule:** All participants described their strict eating and drinking schedule, most often consisting of cornstarch mixed with water, or Kool-Aid, consumed every 2 to 4 hours. Participants also reported needing to snack on carbohydrates like pretzels and crackers. Participants described their life as “living by a clock” (**Table 2, Quote 2**).

Caregivers relied on the use of alarms to notify when their child needed cornstarch. One reported they do not leave their child in the care of others due to the nature of their dietary management.

Participants also described the consequences of missing or delaying a dose of cornstarch, even by a few minutes; these included blood sugar levels dropping to hypoglycemic ranges, seizures, fainting, and hospitalization. One participant said managing GSDIa is “like travelling in a high-speed car with only partial control. It is extremely easy to crash. Years of effort can be erased quickly.”

Cornstarch consumption, usually required throughout the night, was described by 7 caregivers and 7 participants living with GSDIa. Overnight feeding regimes consisted of using a feeding tube or waking up to drink cornstarch. Participants reported a lack of sleep and consequently feeling exhausted during the day (**Table 2, Quote 3**). One participant also detailed how consuming a large amount of cornstarch before going to sleep means they feel uncomfortable and often cannot get to sleep for “hours.”

Participants reported experiencing nausea after consuming cornstarch as well as other problems they attributed to their dietary requirements, including gas and irritation, and weight gain. Participants also reported issues with exercise, being insulin insensitive, and the risk of developing diabetes.

Many participants described the use of feeding tubes such as a gastrostomy tube to manage their GSDIa. Participants had conflicting views on this; one found it made things easier and another indicated being “highly embarrassed” by it. One caregiver reported their child’s liver significantly reduced in size after having a gastrostomy tube while another caregiver commented on the increased risk of infection through gastrostomy tube use.

**Desire for treatment**: Caregivers and participants living with GSDIa expressed a strong desire for a treatment. Participants were not concerned about what this treatment was but had a need for it to improve their quality of life, both physically and mentally (**Table 2, Quote 4**).

### Experience of Healthcare Services

**Diagnostic journey:** Most participants discussed their diagnosis story. Participants living with GSDIa all reported being diagnosed as an infant. Caregivers elaborated on their child’s diagnosis journey. Several caregivers described that before their child’s diagnosis, they could tell their child was “uniquely different” because they were sick very often, constantly needed feeding, “never seemed comfortable and very difficult to console.” One caregiver described “pushing and shoving” the doctor for an answer until they received a diagnosis. One caregiver described the very chaotic events of their child’s diagnosis: their child unconscious in the car journey to the hospital and being met with panic once at the hospital.

Once diagnosed, people with GSDIa were given a prognosis of 1 to 2 years of life expectancy. The participants in this study described “defying the odds,” living to go on to university, have jobs, get married, and have children (**Table 2, Quote 5**).

**Experience with healthcare professionals:** Participants described the lack of knowledge of GSDIa among the healthcare professionals (HCPs) they encountered. It was difficult to find a doctor who could manage their condition, having to go “from doctor after doctor, each not having answers.” Participants described how doctors’ lack of GSDIa knowledge made it harder to deal with their condition. One participant described some of the HCP reactions they encountered (**Table 2, Quotes 6 and 7**).

Participants reported other challenging experiences with HCPs, including misdiagnosis, and many HCPs refused to listen and often ignored participants trying to explain what needed to be done for their care. One participant said their friend with GSDIa died because they did not receive the right care at a hospital.

Participants reported having to fight to get the care they needed and videotaping their child to prove there was something wrong. One caregiver was told by an HCP that their baby was fine, and they were exaggerating. Caregivers described being denied help on multiple occasions.

Participants complained about the lack of communication, lack of hope, feeling let down by HCPs, and the absence of a support system for individuals with GSDIa (**Table 2, Quotes 8 and 9**).

Participants also spoke of some positive experiences with their healthcare system. Many discussed the great medical care they received with doctors giving them hope and encouragement, a feeling of safety, and being a good friend. Caregivers expressed gratitude for the doctors and said they saved their child’s life (**Table 2, Quote 10**).

### Impacts of Living With or Caring for Someone With GSDIa

The burden of GSDIa on individuals was substantial, including frequent admissions to hospital. Participants described being in hospital for varying, often extended, periods of time (**Table 2, Quote 11**). Reasons for hospitalization included experiencing severe blood sugar lows, viruses or infections, and changes in responses to cornstarch. One participant suggested they were in hospital “every other month.”

**Emotional impacts**: Individuals living with GSDIa reported a large range of emotional impacts (**Table 3**), including stress and anxiety around their disease, as well as issues around body image (**Table 2, Quotes 12 and 13**).

**Table 3.** Participant Reported Emotional Impacts

**Emotion**	**Participant With GSDIa (n = 11), n (%)**	**Caregiver-Reported Impacts on Child (n = 8), n (%)**
Mental health	4 (36)	0 (0)
Body image issues	4 (36)	0 (0)
Stress	2 (18)	0 (0)
Anxiety	4 (36)	0 (0)
Desire for better life	8 (73)	1 (13)
Worry and fear	5 (45)	0 (0)
Frustration and anger	5 (45)	3 (38)
Upset	2 (18)	2 (25)
Embarrassment	3 (27)	0 (0)
Suicide	1 (9)	1 (13)
Hiding feelings	2 (18)	0 (0)
Positive attitude	4 (36)	3 (38)
Desire to help others	3 (27)	0 (0)

*Note*: Included in the table if reported by ≥2 participants.

Other feelings reported included fear, mainly around the risk of death, frustration, upset, feeling like a burden, embarrassment surrounding feeds, grief, and anger at the world. Two participants reported feeling suicidal in the past.

**Figure 1.**
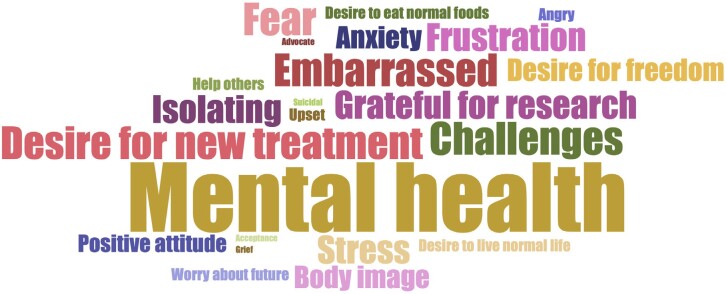
Word Cloud of the Emotions Spontaneously Reported by Participants Living With GSDIa

Two participants discussed having to hide their true feelings to disguise how terrified they are. Participants described desiring spontaneity and freedom from planning, to live a normal life, and to eat the same as others. Participants also said having GSDIa brings about feelings of isolation and loneliness. The emotional experiences of participants living with GSDIa are summarized in the word cloud (**Figure 1**). A word cloud was used to illustrate the data because the data collection was open ended and essentially unstructured.

The size of each word corresponds to the number of times participants reported them overall.

Some participants managed to keep a positive attitude, feeling hopeful, grateful for the opportunities GSDIa has given them, proud for living with GSDIa and learning to manage it, not letting their disease beat them, and not wanting sympathy. Many participants had a desire to help others, with one participant training to become a healthcare professional and another setting up a mentor platform for individuals with GSDIa.

**Social impacts**: Participants described the considerable social impact of having GSDIa or caring for someone with GSDIa. This included missing out on social events or having to leave them early to eat food. Two participants emphasized the difficulty of not being able to drink alcohol as a student and keeping up with “student culture.” Participants also discussed missing out on sleepovers as they required more preparation, and caregivers were afraid they would not wake up to take their cornstarch. Children with GSDIa were unable to attend summer camps, as no camp directors were willing to take on the responsibility of doing night feeds.

Some participants felt they stood out because of their physical appearance. Caregivers also reported that their child often felt uncomfortable drinking cornstarch in front of others (**Table 2, Quote 14**). Participants described how this was noticed by others and led to bullying (**Table 2, Quote 15**).

People living with GSDIa described the difficulty of it being an invisible disease and the stigma around having a condition, with some saying they were treated differently once people knew of their condition (**Table 2, Quotes 16 and 17**).

Several participants discussed the impact on their romantic relationships. For participants living with GSDIa, they discussed how “wonderful” their partner had been through their experience, and another described how “devastated” their partner would be if they were to die prematurely due to GSDIa. Participants commented on the difficulties of dating and becoming intimate, worrying about their “large appearance,” and the gastrostomy hole being “disturbing and frightening” (**Table 2, Quote 18**).

Positive social impacts were also reported. These included friends being accepting and supportive of their condition. Participants also said they met others with GSDIa which made them feel less alone. One caregiver discussed meeting another family that has a child with GSDIa, and the mothers became “each other’s sounding board and support.”

**Impacts on daily activities**: Most people reported missing out on physical activities due to a lack of energy. Participants described being unable to do activities such as swimming, football, or contact sports due to the gastrostomy tube. Several participants also described the impact of GSDIa on traveling. These included difficulties carrying cornstarch through airport security. Caregivers emphasized the stress and amount of preparation that goes into traveling, which meant foreign travel was often avoided.

**Impacts on the wider family**: The impact on the wider family, particularly siblings without GSDIa, was described by caregivers. Other children could not be given the same attention as their sibling with GSDIa, and parents reported feeling guilty about this. Some siblings were affected by cancelled days out or cancelled vacations. One caregiver commented on missing school concerts. Siblings also experienced an emotional burden, especially if their sibling was admitted to the hospital. Two caregivers discussed how their child struggles with fear and insecurity regarding their sibling with GSDIa.

Caregivers also described the effect of caring for someone with GSDIa on themselves. Emotional impacts included constant worry, tiredness, exhaustion, and anxiety around their child being in the care of others. They also reported feelings of stress, upset, frustration, helplessness, and guilt (**Table 2, Quote 19**).

Caregivers described missing out on social events and rarely going out because of their caregiving responsibilities, as well as due to disrupted sleep. Caregivers also described having strained relationships with others, losing friends, or having few friends, which they attributed to their child’s disease. One caregiver suggested their relationship with their spouse was stronger than ever, having to cope with little sleep and being selfless in prioritizing the needs of their child. However, more participants reported a strain on their relationship due to caring for a child with GSDIa, as they had little time together as a couple. One caregiver reported having attended couples’ counseling.

**Professional, educational, and financial impacts:** Taking cornstarch at work or at school presented problems for participants. Participants reported difficulty managing their doses with a demanding job and were also questioned by colleagues. Caregivers believed GSDIa impacted their child’s education, partly due to the disruption of hospital appointments and their child’s lack of energy.

Participants reported limited job opportunities for individuals with GSDIa with a caregiver describing the job scene for her child as “non-existent.” One participant discussed the missed opportunities and limited career choices they have (**Table 2, Quote 20**).

Many caregivers also reported reducing their work hours to part-time, or missing days of work. Caregivers had to put their career aside and take large reductions in income. One parent reported feeling financial pressure after their partner took a pay cut to work part-time and focus on caring for their child.

**Other impacts**: Participants reported on their experiences of the death of a loved one, either one of their children, or a friend with GSDIa. Three caregivers reported losing a child to GSDIa, which left them, and their families “devastated.” Two of these caregivers described regret that their focus on managing GSDIa meant they missed other signs of their child’s illness. One participant described the impact of losing a young friend they had made via a GSDIa support group, which resonated with them deeply (**Table 2, Quote 21**).

### Thematic Networks

**Figure 2.**
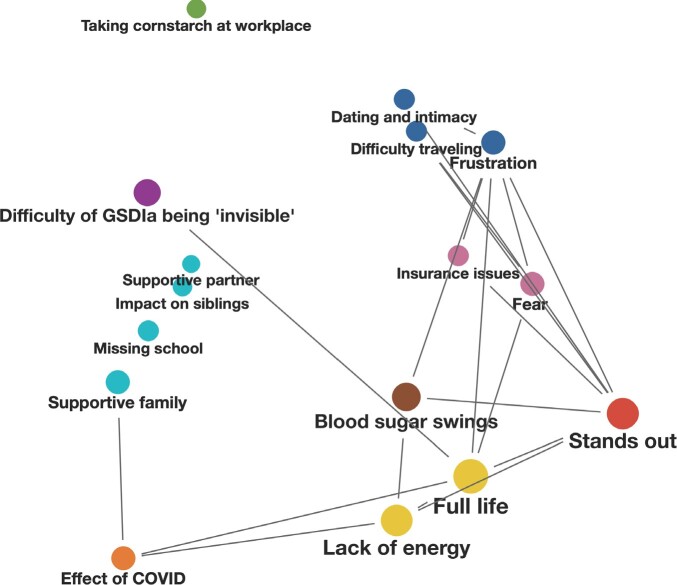
Thematic Network Depicting Symptoms and Impacts of Participants Living With GSDIa

**Figure 2** shows a thematic network displaying the association of symptoms and impacts reported spontaneously by those living with GSDIa. “Full life,” “stands out,” and “lack of energy,” were the most frequently reported by participants living with GSDIa, which were all in close proximity to the code “blood sugar swings” **(Figure 2)**. The code “frustration” was co-reported with codes “stands out,” “dating and intimacy,” “difficulty traveling,” “insurance issues,” and “fear.” The code “taking cornstarch in the workplace” seemed to not be reported in close proximity to any other of the most frequently reported symptoms and impacts.

The network depicts the proximity of codes and themes reported. When generating these figures, *close proximity* was defined as being within the same paragraph. This network analysis was limited to the 2 most frequently reported symptoms and impacts in each category, in order to focus on those considered most meaningful to participants. The size of the code circle represents how frequently the codes were reported in the narratives. Codes joined by a line show where codes are co-reported a minimum of 4 times. Code circles located in close proximity show frequently co-reported codes. The color scheme also depicts the proximity of reported themes, creating clusters of frequently co-reported codes.

## DISCUSSION

This report describes a qualitative study to explore the experiences of people living with or caring for someone with GSDIa. The qualitative data that were captured consisted of a series of open-ended narrative accounts of the impact of the disease. Participants were encouraged to write about what was important to them and were given only very basic example prompts as guidance. Participants were not directed in any other way. These narratives allowed for the collection of contextualized data, which can help us to better understand the lived experience of patients with GSDIa.

Participants reported a range of symptoms that affected day-to-day life. The constant need for cornstarch every 2 to 4 hours was a very significant concern for participants. The intense feeding schedule meant participants had to plan their days carefully around it and were unable to do more spontaneous activities. This issue was often returned to in the narratives. Participants were also very aware of the risks of not taking their cornstarch routinely, such as hypoglycemic episodes or hospitalizations. The nocturnal feeding requirements meant participants’ sleep was interrupted, leading to constant tiredness. The need to manage GSDIa around the clock mirrors previous findings in the literature, where the US Food and Drug Administration conducted interviews with caregivers of individuals with GSDIa.^25^

The lack of knowledge about GSDIa among HCPs was a commonly reported theme. Some found it difficult to find a doctor with expertise to treat GSDIa, others were often misdiagnosed, and many described having to educate HCPs on how to treat their condition when they were hospitalized. Some participants described negative experiences with HCPs, such as having to fight for health care and having their concerns dismissed. However, others reported positive experiences and said they felt well cared for and supported by their HCPs.

Participants reported a range of impacts on their quality of life related to their experience of living with or caring for someone with GSDIa. The emotional impact was one of the biggest, with participants living with GSDIa reporting an effect on their mental health. Some of the mental health impact was related to their body image, with concerns about their short stature or being overweight. A study by Flanagan et al found participants with GSDIa had low body esteem, and concerns about weight and height were commonly reported.^26^ In the current study, this was also cited as a reason for problems with romantic relationships.

Participants described stress and anxiety related to managing their condition and the inherent risks associated with it. Caregivers also reported a considerable emotional impact, with the most common being constant worry and fear, anxiety and stress, tiredness, and exhaustion.

Several social impacts were reported, such as avoiding social activities due to dietary requirements or not drinking alcohol, and missing out on sleepovers or camping trips because caregivers could not leave the responsibility of ensuring timely night feeds to others. The reports are aligned with those from participants in the FDA interviews who said the social impact (making friends, going out with friends, going to college, and dating) was one of the most frustrating aspects of GSDIa.^27^

This study provided novel insights into the impact of GSDIa on individuals living with the condition and their caregivers. Participants reported a wide range of issues in their narratives, which may not have been captured if they had taken part in a semi-structured interview format. Participants shared sensitive information about their experiences and appeared to be open and forthcoming in their narrative descriptions. Many participants reported recognizing the importance of contributing to knowledge about the impact of GSDIa on individuals and their families in this under-researched aspect of the disease and were grateful for the opportunity to tell their story of living with or caring for someone with GSDIa. Participants were given a substantial period of time to complete their narratives, which enabled them to complete the task at their own convenience and to give it considerable thought.

Our informal PubMed searches identified very few existing studies that used a narrative approach to capture essentially unprompted data from study participants and then analyzed the written narratives using qualitative methods. Some studies looked at issues that may be difficult to discuss in an interview, such as bereavement,^28^ and one study used the approach to explore experience of endometriosis.^29^ Other studies employed a written narrative method^30–32^; however, they posed open-ended questions rather than using a fully open-ended narrative approach like we did. The advantage of the open-ended approach is that researchers do not constrain the issues that are discussed and also avoid potential bias introduced by the interviewer-participant interaction. The method allows participants to write at their convenience and to provide as much or little detail as they wish. These advantages make the open-ended narrative approach particularly suitable for the rare-and ultra-rare-disease context.

### Limitations

There were some limitations to consider in this study. First, using the open-ended narrative approach meant that participants could write about issues that were idiosyncratic and therefore not generalizable. However, thematic analysis allowed the team to extract and summarize relevant content from the narratives, even when descriptions were very specific. It was possible that this method could disadvantage people who found it difficult to express themselves by writing. Additionally, without interviewer questions and prompts, the narrative accounts could describe anything, so the researchers were not able to focus the discussion, which could be a challenge to the analysis and interpretation of the results. Furthermore, the recruitment method of identifying participants only through patient associations meant we may not have reached a broad group of patients outside the GSDIa community who may have different experiences of living with GSDIa. Despite this limitation, we were able to recruit a diverse sample for such a rare disease.

### Implications

The findings from this study highlight the importance of truly listening to individuals with a rare disease, who are the experts in their own disease. For clinical practice, this underscores the need for HCPs, particularly nonspecialists, to recognize and validate patient experiences, ensuring that treatment plans and emergency management are developed in collaboration with those living with the condition.

## CONCLUSIONS

It is clear from the challenges reported by participants that there is a significant unmet need among individuals with GSDIa and their caregivers, much of which is associated with the burden of the cornstarch regimen. There is also clearly an unmet need in participants’ healthcare experiences, including difficulties receiving diagnosis or support, challenging encounters with healthcare professionals, and the absence of new or improved treatments or management for GSDIa.

Despite study limitations, this research recruited a large sample of people affected by this rare disease. These methods may be particularly well-suited for better understanding the impact of a rare condition on people and their caregivers. It is hoped that increased understanding of GSDIa could help individuals feel more supported in the management of their condition. Future research should focus on creating more opportunities for individuals with rare diseases to share their experiences in unstructured formats, allowing for deeper insights into their daily challenges and unmet needs. Such qualitative approaches could enhance understanding of the lived experience of GSDIa and other rare diseases and inform more patient-centered care strategies.

### Ethics Approval and Consent to Participate

The protocol was reviewed and approved by the WCG IRB-Western Connexus Group Independent Review Board (IRB No. 1312610).

### Availability of Data and Materials

The data sets used and analyzed during the current study are available from the corresponding author on reasonable request.

### Disclosures

E.K. is currently employed by Moderna. The work presented here was conducted while employed at Ultragenyx and was not influenced by the author’s current employer. I.F. was an employee of the University of Connecticut at the time this work was conducted.
